# Supplementary material for: A correlation analysis between nutritional status and physical function in the elderly with pulmonary tuberculosis
Source: PLoS One. 2025 Sep 8;20(9):e0331700. doi: 10.1371/journal.pone.0331700 (PMC12416726; doi:10.1371/journal.pone.0331700)
Supplement: S2 Table — (DOCX) [file pone.0331700.s002.docx]

**Patients consent form**

| Dear Patient:  You will be invited to participate in a study hosted by the Public Health Clinical Center of ChengDu. This is a study to evaluate the nutritional status of the elderly with pulmonary TB using the Mini Nutritional Assessment (MNA) and explore the relationship between their nutritional status and physical function.  This informed consent form provides you with information to help you decide whether or not to participate in this clinical research study. Your participation in this study is voluntary. This study was approved by the Medical Ethics Committee of Public Health Clinical Centre of Chengdu (NO YJ-K2023-20-01). If you agree to participate in this study, please see the instructions below:  **Study Purpose:** Malnutrition is an important risk factor for the development of tuberculosis (TB) . The prevalence of malnutrition in TB patients ranges from 38.3% to 75% in different countries , while the risk of malnutrition in the elderly of pulmonary TB is as high as about 62.7%. Previous studieshave shown that malnourished elderly TB patients are more prone to symptoms such as emaciation, immunocompromise, debilitation, and even cachexia, which can easily increase the incidence of adverse events such as falls in patients and seriously affect their prognosis. Physical function is an important predictor of health status in elderly patients, as well as their nutritional risk. However, there are fewer studies on the relationship between nutritional status and physical function in the elderly of pulmonary TB. Therefore, this study investigated the nutritional status of the elderly of pulmonary TB using the Mini Nutritional Assessment (MNA) and analyzed the relationship between nutritional status and physical function, providing a reference basis for improving the health of the elderly of pulmonary TB and disease regression.  **Study Method:** This is a cross-sectional study and you will be invited to complete a questionnaire (including general information, nutrition, balance) during your hospitalisation after you have agreed to take part in this study. The questionnaire will take approximately 5 minutes to complete. At the same time, you will take part in Timed Up and Go testt and Five-Times-Sit-to-Stand Tes. The study guarantees that no personal information about any participant will ever be disclosed. All study information will be used only for research and to improve clinical care. This study will not interfere with your normal treatment or life, so please fill in the questionnaire as you really think and do.  **Signature of Informed Consent:**  I have read this informed consent form and my doctor___________ has explained the purpose, content, risks and benefits of this clinical trial to me in detail and answered all of my questions, I understand this clinical study and I volunteer to participate in this study.  Signature of the subject:_____________________  Date:  Signature of the researcher:_____________________  Date: |
| --- |

**Mini Nutritional Assessment (MNA) Record Form**

| 1. Nutritional Screening | **Score** |
| --- | --- |
| 1. Has food intake declined over the past 3 months due to loss of appetite, digestive problems, chewing or swallowing difficulties?   0=Severe loss of appetite  1=Moderate loss of appetite  2=Normal appetite |  |
| 1. Weight loss during the last 3 months   0=Greater than 3 kg  1=1 to 3 kg  2=No weight loss  3=Unknown |  |
| 1. Mobility   0=Bed or chair bound  1=Able to get out of bed/chair but does not go out  2=Goes out |  |
| 1. Has there been any significant psychological stress or acute disease in the past 3 months?   0=Yes  1=No |  |
| 1. Neuropsychological problems   0=Severe dementia or depression  1=Mild dementia  2=No psychological problems |  |
| 1. Body Mass Index (BMI) (kg/m²)   0=Less than 19  1=19 to less than 21  2=21 to less than 23  3=23 or greater |  |
| creening score (subtotal maximum 14): >12 indicates normal (no risk of malnutrition), no further assessment required; <11 suggests possible malnutrition, please continue with the following assessment. | |
| 1. General Assessment | **Score** |
| 1. Lives independently (no nursing care or hospitalization)?   0=No  1=Yes |  |
| 1. Takes more than three prescription drugs per day?   0=No  1=Yes |  |
| 1. Pressure sores or skin ulcers?   0=No  1=Yes |  |
| 1. 10. How many full meals does the patient eat daily? |  |
| 0=No  1=Yes |  |
| 1. Protein intake:   *At least one serving of dairy products per day? A) Yes B) No  *Eggs two or more times per week? A) Yes B) No  *Meat, fish, or poultry daily? A) Yes B) No  0.0= 0 or 1 "Yes"  0.5= 2 "Yes"  1.0= 3 "Yes" |  |
| 1. Consumes two or more servings of vegetables or fruits daily?   0=No  1=Yes |  |
| 1. Daily fluid intake (water, juice, coffee, tea, milk, etc.):   0.0= Less than 3 cups  0.5= 3 to 5 cups  1.0= More than 5 cups |  |
| 1. Feeding ability:   0=Unable to eat independently  1=Some difficulty eating independently  2=Fully independent eating |  |
| 1. Self-assessment of nutritional status:   0=Malnourished  1=Uncertain  2=Well-nourished |  |
| 1. Compared to peers of the same age, how do you assess your health status?   0.0=Not so good  0.5=Don't know  1.0=Good  2.0=Better |  |
| 1. Mid-arm circumference (cm):   0.0=Less than 21  0.5=21 to 22  1.0=22 or greater |  |
| 1. Calf circumference (cm):   0=Less than 31  1=31 or greater |  |
| General assessment score (subtotal maximum 16):  Nutritional screening score (subtotal maximum 14):  MNA total score (maximum 30): |  |
| MNA classification criteria:  A total score ≥24 indicates good nutritional status  A total score of 17~24 indicates a risk of malnutrition  A total score <17 indicates malnutrition |  |

**Berg Balance Scale (BBS)**

| Item | Instruction | Score |  |
| --- | --- | --- | --- |
| 1. Sitting to Standing | Try to stand up without using hands for support | 4 Stands up without support and remains stable  3 Stands up with hand support and remains stable  2 After several attempts, stands up with hand support  1 Needs minimal assistance to stand or stabilize  0 Needs moderate or maximal assistance to stand |  |
| 2. Standing Independently | Please stand independently for 2 minutes | 4 Safely stands independently for 2 minutes  3 Stands for 2 minutes under supervision  2 Stands independently for 30 seconds  1 Needs several attempts to stand independently for 30 seconds  0 Unable to stand independently for 30 seconds  (If the patient can safely stand independently for 2 minutes, the "Sitting Independently" item is scored full points, proceed directly to item 4) |  |
| 3. Sitting Independently | Sit with arms crossed for 2 minutes (back unsupported, feet on the ground or on a low stool) | 4 Sits safely without assistance for 2 minutes  3 Sits for 2 minutes under supervision  2 Sits independently for 30 seconds  1 Sits independently for 10 seconds  0 Needs support to sit for 10 seconds |  |
| 4. Standing to Sitting | Please sit down | 4 Needs minimal assistance (hand support) to sit down safely  3 Needs hand control to sit down slowly  2 Needs to lean back against the chair to control sitting down  1 Can sit down independently but cannot control the descent  0 Needs assistance to sit down |  |
| 5. Transfer: Bed to Chair | Transfer from bed to chair | 4 Transfers safely with minimal use of hands  3 Transfers safely with hand support  2 Transfers with verbal cues/supervision  1 Needs assistance from one person to transfer  0 Needs assistance from two people to transfer/supervise |  |
| 6. Standing with Eyes Closed | Stand with eyes closed for 10 seconds | 4 Safely stands with eyes closed for 10 seconds  3 Stands with eyes closed for 10 seconds under supervision  2 Stands with eyes closed for 3 seconds  1 Unable to stand with eyes closed for 3 seconds but can stand safely  0 Needs assistance to prevent falling |  |
| 7. Standing with Feet Together | Stand with feet together without support | 4 Safely stands with feet together for 1 minute  3 Stands with feet together for 1 minute under supervision  2 Can stand with feet together but cannot maintain for 30 seconds  1 Needs assistance to bring feet together and can maintain for 15 seconds  0 Needs assistance to bring feet together and cannot maintain for 15 seconds |  |
| 8. Standing position, upper limb forward | Raise the upper limb to 90 degrees and extend the fingers as far forward as possible (when the upper limb is at 90 degrees, the doctor places a soft ruler on the end of the fingers without the fingers touching the ruler, and the fingers are extended forward as far as possible when the patient is leaning forward at maximum. Reach forward with both hands as far as possible to avoid body rotation) | 4 Can safely reach forward 25 cm  3 Can reach forward 12 cm  2 Can reach forward 5 cm  1 Can reach forward under supervision  0 Needs external support/loses balance while reaching forward |  |
| 9. Picking Up an Object from the Floor While Standing | Pick up a slipper/object from the floor in front of the feet while standing | 4 Can safely and easily pick up the slipper  3 Can pick up the slipper under supervision  2 Cannot pick up the slipper but can maintain balance independently within 2-5 cm of the object  1 Cannot pick up the slipper, requires supervision during attempts  0 Unable to attempt, requires assistance to prevent loss of balance or falling |  |
| 10. Turning to Look Behind | Turn to the left to look behind, then turn to the right to look behind (the doctor observes directly from behind the patient and encourages turning) | 4 Can look behind on both sides with good weight shift  3 Can look behind on one side with less weight shift on the other side  2 Can only look behind on one side but maintains good balance  1 Requires supervision while turning  0 Needs assistance to prevent loss of balance or falling |  |
| 11. Turning 360 Degrees | Turn 360 degrees clockwise, pause, then turn 360 degrees counterclockwise | 4 Can safely turn 360 degrees in ≤4 seconds  3 Can turn 360 degrees in one direction in ≤4 seconds  2 Can safely turn 360 degrees but slowly  1 Requires close supervision or verbal cues  0 Needs assistance |  |
| 12. Alternating Foot on Step/Stool | Alternately place each foot on a step/stool without support. | 4 Can safely and independently alternate feet 4 times within 20 seconds  3 Can independently alternate feet 4 times in >20 seconds  2 Can alternate feet 2 times under supervision (no assistance needed)  1 Needs minimal assistance to alternate feet >1 time  0 Needs assistance to attempt, to prevent falling |  |
| 13. Standing with One Foot in Front | Step one foot forward. If unable to step directly forward, step as far forward as possible, with the heel of the front foot in front of the toes of the back foot. Step length should exceed foot length, and step width should be approximately equal to the patient's normal step width. | 4 Can independently step forward and backward and maintain for 30 seconds  3 Can independently step forward and maintain for 30 seconds  2 Can take a step and maintain for >30 seconds  1 Needs assistance to step but can maintain for 15 seconds  0 Loses balance while stepping or standing |  |
| 14. Standing on One Leg | Stand on one leg without support for as long as possible | 4 Can stand on one leg independently for >10 seconds  3 Can stand on one leg independently for >5-10 seconds  2 Can stand on one leg independently for >3 seconds  1 Can lift the foot and stand independently but cannot maintain for 3 seconds  0 Unable to attempt, needs assistance to prevent falling |  |
| **Total Score** | | |  |
