# Supplementary material for: A correlation analysis between nutritional status and physical function in the elderly with pulmonary tuberculosis
Source: PLoS One. 2025 Sep 8;20(9):e0331700. doi: 10.1371/journal.pone.0331700 (PMC12416726; doi:10.1371/journal.pone.0331700)
Supplement: Attachment — Submitted filename: Rev1PONE-D-25-13007 MSA.docx [file pone.0331700.s004.docx]

**Review MSA**

1. How to differentiate malnutrition with low and high BMI both are malnutrition, has not been discussed and my affect the result
2. Musculoskeletal disorder status does not excluded? How is the explanation?
3. What about the Tuberculosis profile of the subjects, whether they are sensitive or resistant, extrapulmonary or pulmonary, because it is related to the duration of treatment and will be related to the dependent variable of this research?
4. There is significancy different in age between group (pg 7), I don’t see any discussion about it
5. One of the factors that needs to be considered in the relationship between independent and dependent variables in this study is nutritional intake, which has not been discussed in detail. If there is no data, this can be conveyed in the limitations
6. History of phisical activity or workout could be as a variable that influences the outcomes, has not been discussed.
