# Supplementary material for: A correlation analysis between nutritional status and physical function in the elderly with pulmonary tuberculosis
Source: PLoS One. 2025 Sep 8;20(9):e0331700. doi: 10.1371/journal.pone.0331700 (PMC12416726; doi:10.1371/journal.pone.0331700)
Supplement: Attachment — Submitted filename: Response to reviewers.docx [file pone.0331700.s006.docx]

Dear Editor,

Thank you very much for giving us an opportunity to revise and resubmit our manuscript entitled “A correlation analysis between nutritional status and physical function in the elderly with pulmonary tuberculosis” (Submission ID PONE-D-25-13007) to the **PLOS One**.

We have carefully read your and the reviewer’ comments on our manuscript and would like to thank reviewers for the insightful and helpful critiques.

In response to the reviewer’ comments, we have thoroughly revised our manuscript which marked in red font in the paper and tried our best to revise our manuscript according to reviewer’ detail suggestions.

As a consequence, we believe that the manuscript is considerably strengthened. Below please find our point-by-point response to the reviewers.

Best regards,

Xiaoyi Yang

The Second Department of Tuberculosis

The Public Health Clinical Center of ChengDu

Department of Cardiothoracic Surgery

No.377 jingming Road, jinjiang District, Chengdu, Sichuan 610066, China.

Email: 1365390609@qq.com

**Point-by-point response**

**Journal Requirements:**

1 Please ensure that your manuscript meets PLOS ONE's style requirements, including those for file naming.

Response: Thank you for your comment. We have thoroughly reviewed the manuscript and ensured that it fully complies with PLOS ONE's formatting and style requirements, including the appropriate file naming conventions.

2 Please include captions for your Supporting Information files at the end of your manuscript, and update any in-text citations to match accordingly.

Response: Thank you for your suggestion. We have added a dedicated section titled “Supporting Information” at the end of the manuscript, where each supporting file has been assigned an appropriate title (e.g., S1 Text: Patient Informed Consent Form and Research Questionnaires). In addition, we have updated all in-text citations to ensure consistency with the titles and numbering in the Supporting Information section.

3 Please remove all personal information, ensure that the data shared are in accordance with participant consent, and re-upload a fully anonymized data set.

Response: Thank you for your guidance. We have thoroughly reviewed our dataset and removed all personal information pertaining to the participants to ensure full anonymization. The shared data are now in complete accordance with the participant consent forms. A fully anonymized data set has been re-uploaded as requested. We confirm that no personally identifiable information remains within the dataset, and all necessary steps have been taken to protect participant confidentiality while maintaining the integrity of the research findings.

4 We note that there is identifying data in the Supporting Information file <Supplement material-data.xlsx>. Due to the inclusion of these potentially identifying data, we have removed this file from your file inventory. Prior to sharing human research participant data, authors should consult with an ethics committee to ensure data are shared in accordance with participant consent and all applicable local laws.

Response: Thank you for your comment. We sincerely apologize for the oversight. We have now fully anonymized the dataset by removing all personally identifiable information, in accordance with participant consent and ethical guidelines. A revised and fully anonymized version of the dataset has been re-uploaded. Additionally, prior to data sharing, we have consulted with our institutional ethics committee to ensure compliance with all applicable regulations and participant consent agreements. We confirm that the updated dataset contains no identifying information and is suitable for public sharing.

**Reviewer 1:**

1 Abstract:
- Please state the evaluation methods of physical function in the abstract since it is one of the important parameters of this study.

Response: Thanks for your constructive suggestion. We have stated the the evaluation methods of physical function in the abstract. This study assessed physical function using the Berg Balance Scale (BBS), the Timed Up and Go test (TUG), and the Five-Times-Sit-to-Stand Test (FTSST) on page 1.

- Please rewrite the conclusion in the abstract and the context. The cross-sectional study design cannot prove the causal relationship between parameters so the “indicators” or “predictors” cannot be applied in this study.

Response: Thank you for your question. We agree that the cross-sectional study design cannot prove the causal relationship between parameters. In light of this, we have revised the conclusion in the abstract and the context to reflect only the associations observed in our study.

Below is the revised version of the conclusion in the abstract:

Conclusions: Malnutrition is common in the elderly with pulmonary TB. Nutritional status in these patients is associated with the BBS scores, the TUG, and the FTSST (Page 1).

Below is the revised version of the conclusion in the context:

Our study shows that malnutrition is common in the elderly with pulmonary TB and and highlights a close association between nutritional status and physical function. Compared with well-nourished patients, the abnormal patients showed a significant decrease in the BBS scores, and a significant increase in the TUG and the FTSST. Healthcare professionals should focus on the nutritional status and physical function of patients, identify potential nutritional risk groups through early screening and assessment, and carry out early individualised nutritional interventions to improve patients' physical function and disease prognosis, and improve the quality of life of the elderly with pulmonary TB (Page 15).

2 It is recommended that terminology be used consistently throughout the manuscript. For example, “physical balance function” or “Berg balance scale” or “body balance function”, those words are scattering thorough the context even though authors refer to the same things. The parameters “walking step” and 5 sit up times” are also presented as different terminology in other statements in the manuscript. This is very confusing. Please revise.

Response: Thank you for your comment. We agree that consistent terminology is essential for clarity and scientific rigor. Accordingly, we have carefully reviewed the entire manuscript and standardized the terminology. We now uniformly use “Berg balance scale” (BBS) instead of varying expressions such as “physical balance function” or “body balance function”. Additionally, We uniformly use the Timed Up and Go test (TUG) instead of “walking times” and use the Five-Times-Sit-to-Stand Test (FTSST) instead of “5 sit up times”. We have also revised the entire manuscript accordingly.

3 Methods
- All presentations are not consistent. Please state the operational criteria for categorization of MNA score, Berg Balance Scale, TUG, FTSST which implied in this study. In the results, authors mentioned “well-nourished” and “abnormal-nourished” but in the methods authors categorized as “well-nourished”, “risked malnutrition” and “malnourished”. Please consider revising the writing to ensure consistency with the way other variables are presented in the manuscript.

Response: Thank you for your comment. We have supplemented the text with operational definitions and categorisation criteria for each research tools. In addition, the full scale is included in the appendix. Below is a detailed description of each tool. At the same time, we explained in the methods why the categories of “well-nourished”, “risked malnutrition” and “malnourished” were split into “well-nourished” and “abnormal-nourished” 2 groups for comparison. Based on clinical experience and relevant literature, all TB patients with nutritional risk should be assessed for their nutritional status. Therefore, in our study, patients with MNA scores <24 points (i.e. risked malnutrition: 17-24 and malnourished: <17 points), were categorised as abnormal-nourished group. This is mentioned in the abstract section (Page 1) and the introduction section of the Mini nutritional assessment (Page 4). In addition, we have made additional additions and inserted relevant citations in the operational criteria (Page 4-5).

Mini nutritional assessment (MNA)

The assessment was an internationally recommended nutritional screening tool for elderly patients [19–21]. It consisted of anthropometric indicators (4 entries), overall assessment (6 entries), dietary assessment (6 entries) and subjective rating (2 entries). There were 18 entries with a total score of 30.The judgement criteria was well nourished: ≥24 points, risked malnutrition: 17 to <24 points, malnourished: <17 points. The scale demonstrates effective screening performance for hospitalized elderly patients with chronic diseases [22]. Based on clinical experience and relevant literature [23, 24], all TB patients with nutritional risk should be assessed for their nutritional status. Therefore, in our study, patients with MNA scores <24 points (i.e. risked malnutrition: 17-24 and malnourished: <17 points), were categorised as abnormal-nourished group [25].

**Mini Nutritional Assessment (MNA) Record Form**

| 1. Nutritional Screening | **Score** |
| --- | --- |
| 1. Has food intake declined over the past 3 months due to loss of appetite, digestive problems, chewing or swallowing difficulties?   0=Severe loss of appetite  1=Moderate loss of appetite  2=Normal appetite |  |
| 2. Weight loss during the last 3 months   0=Greater than 3 kg  1=1 to 3 kg  2=No weight loss  3=Unknown |  |
| 3. Mobility   0=Bed or chair bound  1=Able to get out of bed/chair but does not go out  2=Goes out |  |
| 4. Has there been any significant psychological stress or acute disease in the past 3 months?   0=Yes  1=No |  |
| 5. Neuropsychological problems   0=Severe dementia or depression  1=Mild dementia  2=No psychological problems |  |
| 6. Body Mass Index (BMI) (kg/m²)   0=Less than 19  1=19 to less than 21  2=21 to less than 23  3=23 or greater |  |
| creening score (subtotal maximum 14): >12 indicates normal (no risk of malnutrition), no further assessment required; <11 suggests possible malnutrition, please continue with the following assessment. | |
| 1. General Assessment | **Score** |
| 7. Lives independently (no nursing care or hospitalization)?   0=No  1=Yes |  |
| 8. Takes more than three prescription drugs per day?   0=No  1=Yes |  |
| 9. Pressure sores or skin ulcers?   0=No  1=Yes |  |
| 10. 10. How many full meals does the patient eat daily? |  |
| 0=No  1=Yes |  |
| 11. Protein intake:   *At least one serving of dairy products per day? A) Yes B) No  *Eggs two or more times per week? A) Yes B) No  *Meat, fish, or poultry daily? A) Yes B) No  0.0= 0 or 1 "Yes"  0.5= 2 "Yes"  1.0= 3 "Yes" |  |
| 12. Consumes two or more servings of vegetables or fruits daily?   0=No  1=Yes |  |
| 13. Daily fluid intake (water, juice, coffee, tea, milk, etc.):   0.0= Less than 3 cups  0.5= 3 to 5 cups  1.0= More than 5 cups |  |
| 14. Feeding ability:   0=Unable to eat independently  1=Some difficulty eating independently  2=Fully independent eating |  |
| 15. Self-assessment of nutritional status:   0=Malnourished  1=Uncertain  2=Well-nourished |  |
| 16. Compared to peers of the same age, how do you assess your health status?   0.0=Not so good  0.5=Don't know  1.0=Good  2.0=Better |  |
| 17. Mid-arm circumference (cm):   0.0=Less than 21  0.5=21 to 22  1.0=22 or greater |  |
| 18. Calf circumference (cm):   0=Less than 31  1=31 or greater |  |
| General assessment score (subtotal maximum 16):  Nutritional screening score (subtotal maximum 14):  MNA total score (maximum 30): |  |
| MNA classification criteria:  A total score ≥24 indicates good nutritional status  A total score of 17~24 indicates a risk of malnutrition  A total score <17 indicates malnutrition |  |

Berg Balance Scale (BBS)

The scale consisted of 14 balance-related entries as a means of assessing functional body balance in older adults [26]. A 4-point Likert scale (0 indicating the lowest level of functions and 4 indicating the highest) was used, with a total score of 56. 0-20 points (poor balance ability and can only sit in a wheelchair ), 21-40 points (balance ability and can assist with walking), 41-56 points (good balance ability and can walk independently) [27]. The Cronbach's alpha of the scale was 0.864.

**Berg Balance Scale (BBS)**

| Item | Instruction | Score |  |
| --- | --- | --- | --- |
| 1. Sitting to Standing | Try to stand up without using hands for support | 4 Stands up without support and remains stable  3 Stands up with hand support and remains stable  2 After several attempts, stands up with hand support  1 Needs minimal assistance to stand or stabilize  0 Needs moderate or maximal assistance to stand |  |
| 2. Standing Independently | Please stand independently for 2 minutes | 4 Safely stands independently for 2 minutes  3 Stands for 2 minutes under supervision  2 Stands independently for 30 seconds  1 Needs several attempts to stand independently for 30 seconds  0 Unable to stand independently for 30 seconds  (If the patient can safely stand independently for 2 minutes, the "Sitting Independently" item is scored full points, proceed directly to item 4) |  |
| 3. Sitting Independently | Sit with arms crossed for 2 minutes (back unsupported, feet on the ground or on a low stool) | 4 Sits safely without assistance for 2 minutes  3 Sits for 2 minutes under supervision  2 Sits independently for 30 seconds  1 Sits independently for 10 seconds  0 Needs support to sit for 10 seconds |  |
| 4. Standing to Sitting | Please sit down | 4 Needs minimal assistance (hand support) to sit down safely  3 Needs hand control to sit down slowly  2 Needs to lean back against the chair to control sitting down  1 Can sit down independently but cannot control the descent  0 Needs assistance to sit down |  |
| 5. Transfer: Bed to Chair | Transfer from bed to chair | 4 Transfers safely with minimal use of hands  3 Transfers safely with hand support  2 Transfers with verbal cues/supervision  1 Needs assistance from one person to transfer  0 Needs assistance from two people to transfer/supervise |  |
| 6. Standing with Eyes Closed | Stand with eyes closed for 10 seconds | 4 Safely stands with eyes closed for 10 seconds  3 Stands with eyes closed for 10 seconds under supervision  2 Stands with eyes closed for 3 seconds  1 Unable to stand with eyes closed for 3 seconds but can stand safely  0 Needs assistance to prevent falling |  |
| 7. Standing with Feet Together | Stand with feet together without support | 4 Safely stands with feet together for 1 minute  3 Stands with feet together for 1 minute under supervision  2 Can stand with feet together but cannot maintain for 30 seconds  1 Needs assistance to bring feet together and can maintain for 15 seconds  0 Needs assistance to bring feet together and cannot maintain for 15 seconds |  |
| 8. Standing position, upper limb forward | Raise the upper limb to 90 degrees and extend the fingers as far forward as possible (when the upper limb is at 90 degrees, the doctor places a soft ruler on the end of the fingers without the fingers touching the ruler, and the fingers are extended forward as far as possible when the patient is leaning forward at maximum. Reach forward with both hands as far as possible to avoid body rotation) | 4 Can safely reach forward 25 cm  3 Can reach forward 12 cm  2 Can reach forward 5 cm  1 Can reach forward under supervision  0 Needs external support/loses balance while reaching forward |  |
| 9. Picking Up an Object from the Floor While Standing | Pick up a slipper/object from the floor in front of the feet while standing | 4 Can safely and easily pick up the slipper  3 Can pick up the slipper under supervision  2 Cannot pick up the slipper but can maintain balance independently within 2-5 cm of the object  1 Cannot pick up the slipper, requires supervision during attempts  0 Unable to attempt, requires assistance to prevent loss of balance or falling |  |
| 10. Turning to Look Behind | Turn to the left to look behind, then turn to the right to look behind (the doctor observes directly from behind the patient and encourages turning) | 4 Can look behind on both sides with good weight shift  3 Can look behind on one side with less weight shift on the other side  2 Can only look behind on one side but maintains good balance  1 Requires supervision while turning  0 Needs assistance to prevent loss of balance or falling |  |
| 11. Turning 360 Degrees | Turn 360 degrees clockwise, pause, then turn 360 degrees counterclockwise | 4 Can safely turn 360 degrees in ≤4 seconds  3 Can turn 360 degrees in one direction in ≤4 seconds  2 Can safely turn 360 degrees but slowly  1 Requires close supervision or verbal cues  0 Needs assistance |  |
| 12. Alternating Foot on Step/Stool | Alternately place each foot on a step/stool without support. | 4 Can safely and independently alternate feet 4 times within 20 seconds  3 Can independently alternate feet 4 times in >20 seconds  2 Can alternate feet 2 times under supervision (no assistance needed)  1 Needs minimal assistance to alternate feet >1 time  0 Needs assistance to attempt, to prevent falling |  |
| 13. Standing with One Foot in Front | Step one foot forward. If unable to step directly forward, step as far forward as possible, with the heel of the front foot in front of the toes of the back foot. Step length should exceed foot length, and step width should be approximately equal to the patient's normal step width. | 4 Can independently step forward and backward and maintain for 30 seconds  3 Can independently step forward and maintain for 30 seconds  2 Can take a step and maintain for >30 seconds  1 Needs assistance to step but can maintain for 15 seconds  0 Loses balance while stepping or standing |  |
| 14. Standing on One Leg | Stand on one leg without support for as long as possible | 4 Can stand on one leg independently for >10 seconds  3 Can stand on one leg independently for >5-10 seconds  2 Can stand on one leg independently for >3 seconds  1 Can lift the foot and stand independently but cannot maintain for 3 seconds  0 Unable to attempt, needs assistance to prevent falling |  |
| **Total Score** | | |  |

Timed Up and Go test (TUG)

The test is recommended by the American Geriatrics Society as an indicator for assessing the patient's balance and walking function, especially for the elderly[28]. Test method: During the test, the patients wore their usual shoes, sat on a chair with armrests and a backrest, leaned their body on the back of the chair, and placed their hands on the armrests (seat height about 45cm, armrest height about 20cm). Stick or place a visible marker on the floor 3 metres away from the chair. When the command "start" was heard, the patient stood up firmly, followed the usual walking gait, walked towards the marker, turned around, returned to the chair, sat down, and leaned against the back of the chair. The researcher recorded the time it took the patient to complete the entire walking process, with the shorter time indicating the patient's ability to walk and balance better. The criteria were as follows: ＜10s was well, suggesting free movement; ≥10s was adverse, suggesting impaired movement [29].

Five-Times-Sit-to-Stand Test (FTSST)

This test, which recorded the time it took the subject to repeatedly stand up and sit down five times from a chair, was commonly used to assess lower limb strength and balance in the elderly [30]. Test method: The patients crossed their arms in front of their chests, looked straight ahead, and stood up and sat down 5 times as fast as they could from a 46cm high chair. All patients repeated the test 3 times with a 1 min break in between, and the average of the 3 times was taken as the final test result. Finally, the average test time for all patients was 10s. Thus, Time ≥10s was considered risk of falling; <10s was considered good balance and no risk [31].

- Please indicate the citation for the cut-off points of each score
Response: Thank you for your comment. We have added detailed citations for the cut-off points of each scoreto each research tools (MNA score, Berg Balance Scale, TUG, FTSST). (Page 4-5)

- Please state the statistical test for normalization testing

Response: Thank you for your suggestions. We have described the statistical methods for normality testing in the statistical analysis. All continuous variables were tested for normality using the Shapiro-Wilk test (α=0.05). Variables that conformed to normal distribution (Berg Balance Scale) were described using mean ± standard deviation, and t-tests were used for comparisons between groups. Variables that did not fit the normal distribution (Timed Up and Go test and Five-Times-Sit-to-Stand Test) were described by median [IQR], and comparisons between groups were made using the two related samples rank-sum test. Count data were described by n (%), and comparisons between groups were made using the χ² test. Correlations were analyzed using spearman's correlation analysis, and a difference of P<0.05 was considered statistically significant. (Page 6)

4 Results
- Please describe any concurrent infections (HIV, opportunistic infections, etc.) and details of comorbidities since all those factors involved in nutritional status and clinical outcomes

Response: Thank you for your comment. This is indeed a very important and worthwhile question. In this study, we did not systematically collect specific information about co-infections and comorbidities due to the limitations of the scope of data collection. This is a limitation of this study. In the revised manuscript, we will add a description of this limitation in the “Study limitations” section, and note that future studies should include such information more comprehensively to further clarify its impact on nutritional and clinical outcomes. Below is a detailed explanation of this issue.

Additionally, due to limitations in our data collection scope, we did not systematically gather specific information on co-infections and complications. Future studies could incorporate such data more comprehensively to better assess their impact on nutritional and clinical outcomes. (Page 14)

- A set of data must be presented either narrative or table but NOT both. Please rewrite the results and tables to NOT duplicate the same things.
Response: Thanks to your comments. We have adjusted the article to ensure that the same set of data is presented only in textual descriptions or tables to avoid repetitive expressions. Tables are used to present specific data, while the text only summarizes the main findings and does not repeat specific values to keep the presentation concise and logical.

- Please indicate the statistical tests applied for the P-value as a footnote (under each table)

Response: We have indicated the statistical tests performed on the p-values as footnotes under each table.

Table 1: There are no statistical tests in Table 1, only descriptive.

Table 2: *P* values represented whether the comparison between the two groups is significant. Count variables were tested by chi-square test. Continuous variables were tested with t-test and the two related samples rank-sum test. **P*<0.05 was considered statistically significant.

Table 3: *P* values represented whether the comparison between the two groups is significant. *P* values were derived from the t-test (t) for normally distributed variables and the two related samples rank-sum test (Z) for non-normally distributed variables. **P*<0.05 was considered statistically significant.

Table 4: *P* values represented Spearman's test. **P*<0.05 was considered statistically significant.

- Please carefully check and specify the units of the parameters mentioned in the manuscript. There are missing units for the numbers in the results and tables.

Response: Thanks to your reminder, we have double-checked all the parameter units mentioned in the manuscript and added the missing units in the previous results and tables. It was ensured that all figures were accompanied by the correct units to ensure the accuracy and readability of the data.

- Again, all terminology must be used consistently throughout the manuscript

Response: We appreciate your comment. We have thoroughly checked and revised the manuscript to ensure all terminology is used consistently throughout.

5 The discussion section should be improved. In my opinion, the authors tend to jump between variables without a clear structure and do not sufficiently refer to the actual findings of this study. A more focused and cohesive interpretation of the results is recommended. For example, abnormal BMI found in this study was 46.7% in the well-nourished groups and 45.7% in the abnormal-nourished groups, but authors stated “…the elderly in pulmonary TB with abnormal BMIs have worse nutritional status”. Another example, “YES” drinking status in the well-nourished groups was 26.65% and in in the abnormal-nourished groups was 13.7%, but author discussed “…alcohol consumption impairs the immune system, especially immune cells, limits the ability of monocytes to
regulate inflammation, increases inflammation in the patient's body, and increases energy expenditure, leading to a significant increase in the risk of malnutrition”. Please confirm.

Response: Thank you very much for your valuable comments on our manuscript. We fully agree with your suggestion that the Discussion section needs to be further improved and structurally optimized. In response to the problems you have pointed out, we have already carried out a comprehensive sorting and rewriting of the Discussion section, aiming at clearer logic, more focused, and better analyzing and explaining with the actual results of the study (Page 10-13). The revised discussion section is as follows.

*Potential factors influencing the nutritional status of elderly tuberculosis patients.*

We compared the clinical characteristics of elderly tuberculosis patients in the well-nourished group and the abnormal-nourished group, and found significant differences between the two groups in terms of gender, age, alcohol consumption, BMI, and three physical function tests. These differences are discussed in detail as follows:

(1) Gender: In this study, women had poorer nutritional status than men, which was consistent with the results of a large sample survey [35]. This may be due to the fact that metabolism in men's bodies is faster than in women's, and they have a greater ability to absorb nutrients, thus being better nourished.

(2) Age: Our study found that among elderly TB patients (aged >75), the proportion of cases with poor nutritional status was significantly higher than those with good nutritional status. Several factors may contribute to this finding. First, the physical function and metabolism of elderly individuals gradually decline, leading to physiological deterioration that may result in insufficient nutrient intake [36]. Second, tuberculosis patients are prone to high metabolism and energy expenditure, which increases energy consumption and can lead to malnutrition [6]. Furthermore, nutritional risk increases with age, highlighting the need for greater attention to the nutritional risks and malnutrition issues in elderly patients [37]. Lastly, a sample study in China indicated that factors such as age, disease duration, and the presence of chronic diseases were significantly positively correlated with nutritional risk. The older the individual, the higher the likelihood of malnutrition, and this is closely related to the severity of the disease [38].

(3) Alcohol consumption: In traditional beliefs, alcohol consumption, especially excessive drinking, is known to suppress food intake and nutrient absorption. In TB patients, excessive alcohol consumption may lead to inadequate energy intake and nutrient deficiencies, particularly in elderly patients. Research has shown that alcohol consumption is associated with conditions such as low body weight and low serum albumin, both of which are signs of malnutrition [39]. These factors can exacerbate the disease burden in TB patients. However, our study showed that the proportion of alcohol-consuming patients in the abnormal-nourished group (13.7%) was lower than that in the well-nourished group (26.65%). We hypothesize that this phenomenon may reflect the association between alcohol consumption and factors such as better socio-economic conditions, improved mental health, or positive social interactions. For instance, in some social environments, alcohol consumption is often linked to higher income, better healthcare conditions, and stronger social support, which may help elderly individuals maintain better nutritional status [40]. Additionally, alcohol may promote social interactions, alleviate anxiety, and indirectly improve appetite and overall health, thereby helping maintain a better nutritional status [41]. Furthermore, alcohol consumers may pay more attention to the diversity and nutritional balance of their diet, which could contribute to a superior overall nutritional status compared to non-drinkers [42]. However, this result needs to be further validated through additional research to better understand the relationship between alcohol consumption, nutritional status, and tuberculosis treatment.

(4) BMI: According to the "Guidelines for the Appropriate Range of Body Mass Index and Weight Management in the Elderly in China (T/CNSS 021-2023) [43]," the normal range for BMI is 18.5–24.0. A low BMI (<18.5) is typically associated with malnutrition, protein deficiency, muscle atrophy, and other issues, and such patients often face problems like impaired immune function, physical weakness, and poor response to disease treatment. Previous studies have shown that low BMI is a risk factor for TB, as it can lead to weakened immunity, increased mortality, and affect the effectiveness of anti-TB treatment [44-46]. Our study showed that the proportion of low BMI patients in the abnormal-nourished group (31.0%) was significantly higher than in the well-nourished group (22.0%), which is consistent with previous findings. A high BMI (>25), associated with obesity, has an unclear relationship with TB risk. Our study found that the proportion of high BMI patients in the well-nourished group (24.7%) was significantly higher than in the abnormal-nourished group (14.7%). There is also evidence suggesting that a very high BMI may affect the treatment efficacy of TB [6], but the specific mechanisms involved still require further investigation.

**Reviewer 2:**

1 How to differentiate malnutrition with low and high BMI both are malnutrition, has not been discussed and my affect the result.

Response: Thanks for your constructive suggestion. Regarding how to differentiate malnutrition with low and high BMI both are malnutrition, we have added a detailed discussion on this point in the revised manuscript (Page 12-13). The specific content is as follows:

(4) BMI: According to the "Guidelines for the Appropriate Range of Body Mass Index and Weight Management in the Elderly in China (T/CNSS 021-2023) [43]," the normal range for BMI is 18.5–24.0. A low BMI (<18.5) is typically associated with malnutrition, protein deficiency, muscle atrophy, and other issues, and such patients often face problems like impaired immune function, physical weakness, and poor response to disease treatment. Previous studies have shown that low BMI is a risk factor for TB, as it can lead to weakened immunity, increased mortality, and affect the effectiveness of anti-TB treatment [44-46]. Our study showed that the proportion of low BMI patients in the abnormal-nourished group (31.0%) was significantly higher than in the well-nourished group (22.0%), which is consistent with previous findings. A high BMI (>25), associated with obesity, has an unclear relationship with TB risk. Our study found that the proportion of high BMI patients in the well-nourished group (24.7%) was significantly higher than in the abnormal-nourished group (14.7%). There is also evidence suggesting that a very high BMI may affect the treatment efficacy of TB [6], but the specific mechanisms involved still require further investigation.

2 Musculoskeletal disorder status does not excluded? How is the explanation?

Response: Thanks for your constructive suggestion. Patients with musculoskeletal disorders were excluded from this study. We added this in the exclusion criteria (Page 14).

3 What about the Tuberculosis profile of the subjects, whether they are sensitive or resistant, extrapulmonary or pulmonary, because it is related to the duration of treatment and will be related to the dependent variable of this research?

Response: Thank you for your valuable comments on the characterization of tuberculosis in the study subjects. The type of TB (sensitive/drug-resistant) and site of lesion (intrapulmonary/extrapulmonary) that you mentioned may indeed affect treatment duration and correlate with the study results. In the study, we included patients with confirmed TB and collected the type of treatment (primary/retreatment) and duration of treatment (<1 year/≥1 year) in a questionnaire. However, due to study design limitations, we did not specifically collect data on drug resistance status and lesion site, which is indeed one of the limitations of this study. We have added this point in the “Study limitations” (see page 14) and will further improve data collection in future studies. Below is a detailed explanation of this issue.

Additionally, due to constraints in data collection, we did not systematically record detailed information on TB drug resistance (sensitive vs. resistant), disease localization (pulmonary vs. extrapulmonary). Future studies could incorporate such data more comprehensively to better assess their impact on nutritional and clinical outcomes.

4 There is significancy different in age between group (pg 7), I don’t see any discussion about it

Response: Thank you for raising this important point. We have now added a detailed discussion regarding the age difference between groups in the revised manuscript (Page 11).

(2) Age: Our study found that among elderly TB patients (aged >75), the proportion of cases with poor nutritional status was significantly higher than those with good nutritional status. Several factors may contribute to this finding. First, the physical function and metabolism of elderly individuals gradually decline, leading to physiological deterioration that may result in insufficient nutrient intake [36]. Second, tuberculosis patients are prone to high metabolism and energy expenditure, which increases energy consumption and can lead to malnutrition [6]. Furthermore, nutritional risk increases with age, highlighting the need for greater attention to the nutritional risks and malnutrition issues in elderly patients [37]. Lastly, a sample study in China indicated that factors such as age, disease duration, and the presence of chronic diseases were significantly positively correlated with nutritional risk. The older the individual, the higher the likelihood of malnutrition, and this is closely related to the severity of the disease [38].

5 One of the factors that needs to be considered in the relationship between independent and dependent variables in this study is nutritional intake, which has not been discussed in detail. If there is no data, this can be conveyed in the limitations

Response: Thank you for your comment. Unfortunately, we did not take into account the variable of nutritional intake. We recognize this as a limitation of our study and note it in the limitations in the study in the manuscript (Page 14) to ensure transparency of the study. It is suggested that future studies incorporating nutritional data will provide a more comprehensive understanding of this relationship.

6 History of phisical activity or workout could be as a variable that influences the outcomes, has not been discussed.

Response: Thank you for your comments. We fully understand the importance of history of phisical activity or workout in influencing study outcomes. However, due to the limitations of the study design, this study did not directly collect data from participants related to history of physical activity. To compensate for this, we employed a variety of standardized physical functioning assessment tools to indirectly reflect the potential impact of these factors on study outcomes.

Specifically, we assessed patients' physical function by three methods: the Berg Balance Scale (BBS), the Timed Up and Go Test (TUG), and the Five-Times-Sit-to-Stand Test (FTSST). These assessment tools not only contributed to a comprehensive understanding of the somatic functional status of elderly TB patients, but also provided a reliable quantitative basis for exploring the relationship between their nutritional status and physical function.

Despite failing to obtain information on participants' exercise history, we believe that changes in variables associated with physical activity levels can be captured to some extent through the assessment of the functional indicators described above. In the discussion section (Page 13-14), we also further explore the potential impact that exercise history may have on the relationship between nutritional status and physical function. Below is a discussion of this section.

(5) The differences in the three physical function tests will be discussed in detail in the following section.

Overall, clinical practice should actively identify risk factors for malnutrition through early screening and assessment, provide targeted preventive measures, reduce risk factors for malnutrition, and maximize good nutritional function to improve the nutritional status of elderly tuberculosis patients.

*The correlation between nutritional status and physical function in elderly*

Physical functional status is an important indicator of health status in the elderly and a significant predictor of malnutrition and nutritional risk. BBS is used to assess physical balance ability; TUG test is used to evaluate the ability of elderly individuals to transition from sitting to standing and walking, typically measuring dynamic balance; and the FTSTS is used to assess lower limb muscle strength and functional independence. The indicators and correlation analysis showed that the elderly in pulmonary TB with poorer nutritional status had a significant decrease in the BBS scores (t=5.854, *p<*0.05), and a significant increase in the TUG (Z=-2.710, *p=*0.007) and the FTSST (Z=-5.590, *p*<0.05), i.e. poorer physical functions in patients with abnormal nutrition compared to well-nourished patients. Previous studies have shown that malnutrition leads to muscle strength loss, decreased bone density, and accelerated joint function deterioration, which negatively impact balance and physical stability. This, in turn, results in longer completion times for the TUG test and poorer performance in the FTSTS test [47-49]. CHRISTA et al. also found that malnutrition was a risk factor for physical dysfunction, with people with abnormal nutrition being four times more likely to develop physical dysfunction than those who are well-nourished [50]. A randomized controlled study in the United States [51] demonstrated a strong association between malnutrition and muscle strength and functional status, indicating that increasing nutrients not only improves nutritional status but also enhances balance and physical function. Meanwhile, the WHO considers nutritional support a key factor in tuberculosis treatment [52]. What’s more, exercise history also has an indirect positive effect on nutritional status. Regular physical activity not only stimulates appetite and boosts metabolism but also enhances muscle mass, which improves nutrient absorption and utilization efficiency. As a result, this leads to better performance in the aforementioned physical function tests in patients [47, 49]. Therefore, healthcare professionals should conduct early screening and assessment of malnutrition, implement individualized nutritional interventions, and provide targeted preventive measures, such as nutritional supplementation and exercise guidance, to reduce risk factors for malnutrition and prevent physical function impairments.
