# Supplementary material for: A correlation analysis between nutritional status and physical function in the elderly with pulmonary tuberculosis
Source: PLoS One. 2025 Sep 8;20(9):e0331700. doi: 10.1371/journal.pone.0331700 (PMC12416726; doi:10.1371/journal.pone.0331700)
Supplement: Attachment — Submitted filename: Response_to_reviewers_auresp_2.docx [file pone.0331700.s007.docx]

Dear Editors,

Thank you very much for giving us an opportunity to revise and resubmit our manuscript entitled “A correlation analysis between nutritional status and physical function in the elderly with pulmonary tuberculosis” (Submission ID PONE-D-25-13007) to the **PLOS One**.

We have carefully read your and the reviewer’ comments on our manuscript and would like to thank reviewers for the insightful and helpful critiques.

In response to the reviewer’ comments, we have thoroughly revised our manuscript which marked in red font in the paper and tried our best to revise our manuscript according to reviewer’ detail suggestions.

As a consequence, we believe that the manuscript is considerably strengthened. Below please find our response to the reviewers.

Best regards,

Xiaoyi Yang

The Second Department of Tuberculosis

The Public Health Clinical Center of ChengDu

Department of Cardiothoracic Surgery

No.377 jingming Road, jinjiang District, Chengdu, Sichuan 610066, China.

Email: 1365390609@qq.com

**Reviewer 1:**

- Almost all issues were clarified; however, some statements are still redundant with the tables presented in the results. The conclusion still overclaimed what was found in this study.

Response: Thanks for your constructive suggestion. We fully agree with the reviewers' comments. To avoid repetition with the table content, we have reviewed the results section sentence by sentence, deleted redundant data repetition, and retained only a summary description of key trends and major findings. The revised text is more concise and highlights the key points. The relevant adjustments are clearly marked in “revision mode” in the revised draft. Additionally, We agree that the original wording may have overstated the study’s findings. Accordingly, we have revised the conclusion to present the results more cautiously, clarifying that our findings suggest an association rather than causation and emphasizing the need for further research. The modified conclusion now reads

“Our study shows that malnutrition is prevalent among in the elderly with pulmonary TB and may be associated with poorer physical function. Compared with well-nourished patients, the abnormal patients tended to have lower BBS scores and higher TUG and FTSST, though further research is needed to confirm causality. These findings highlight the potential importance of nutritional screening and assessment in this population. Early identification of malnutrition and tailored interventions may help improve physical function and quality of life, but additional studies are warranted.” (Page 15)

We hope these revisions address the reviewer’s concern and better reflect the scope of our study’s implications. Thank you for your constructive critique, which has strengthened our manuscript.

**Reviewer 2:**

- I accepted all revision that have been made by authors, despite all limitation this article could be improve reader insight about tuberculosis and it relationship

Response: We sincerely appreciate your time and constructive feedback, which has significantly improved the quality of our manuscript. We are grateful for your acknowledgment of the revisions and your encouraging comment regarding the article’s potential to enhance readers’ understanding of tuberculosis and its associated factors.

As you highlighted, while our study has limitations, we hope it provides valuable insights and serves as a foundation for future research in this field. Thank you once again for your thoughtful review and support. We look forward to contributing further to the scientific discourse on this important topic.
